# Enhancing Hydrogen Storage Properties of MgH_2_ by Transition Metals and Carbon Materials: A Brief Review

**DOI:** 10.3389/fchem.2020.00552

**Published:** 2020-07-02

**Authors:** Ze Sun, Xiong Lu, Farai Michael Nyahuma, Nianhua Yan, Jiankun Xiao, Shichuan Su, Liuting Zhang

**Affiliations:** College of Energy and Power, Jiangsu University of Science and Technology, Zhenjiang, China

**Keywords:** hydrogen storage, MgH_2_, transition metals, carbon materials, cycling performance

## Abstract

Magnesium hydride (MgH_2_) has attracted intense attention worldwide as solid state hydrogen storage materials due to its advantages of high hydrogen capacity, good reversibility, and low cost. However, high thermodynamic stability and slow kinetics of MgH_2_ has limited its practical application. We reviewed the recent development in improving the sorption kinetics of MgH_2_ and discovered that transition metals and their alloys have been extensively researched to enhance the de/hydrogenation performance of MgH_2_. In addition, to maintain the cycling property during the de/hydrogenation process, carbon materials (graphene, carbon nanotubes, and other materials) have been proved to possess excellent effect. In this work, we introduce various categories of transition metals and their alloys to MgH_2_, focusing on their catalytic effect on improving the hydrogen de/absorption performance of MgH_2_. Besides, carbon materials together with transition metals and their alloys are also summarized in this study, which show better hydrogen storage performance. Finally, the existing problems and challenges of MgH_2_ as practical hydrogen storage materials are analyzed and possible solutions are also proposed.

## Introduction

Since the industrial revolution, human society is developing rapidly with continuous improvement in technology and rising demand for energy consumption (Pudukudy et al., 2014; He et al., 2016). Unfortunately, fossil fuels, which play dominate role in promoting the development of world, are not renewable and going to be running out in near future. Besides, the severe environmental problems caused by the excessive exploitation and use of fossil fuels, such as the greenhouse effect, ozone layer depletion, acid rains, and pollution, are damaging and threating the ecological balance of the earth. To mitigate the degradation of the earth, various measures have been taken by scientists to explore renewable and clean alternatives to fossil fuels.

Hydrogen, with its safe, high energy density (142 KJ/kg), environment friendliness, convenient and renewability, is proved to be the most promising sustainable and clean energy to replace fossil energy (Cao et al., 2016; Wan et al., 2020). As an energy carrier, hydrogen is abundant on earth and can be produced from any primary energy fuel: coal, oil, nuclear, natural gas, all sorts of renewable energies, and from grid electricity. Hydrogen also has a huge calorific value of energy, which is three times higher than that of petrol (43 MJ/kg) after combustion. Moreover, the dominating combustion product of hydrogen is clean and non-toxic water. Due to above advantages, hydrogen has received extensive attention from researchers worldwide and has made a rapid progress in recent decades (Winter, 2009; Sadhasivam et al., 2017; Peter, 2018). In order to realize the practical application of hydrogen energy, three challenges need to be conquered presciently, which are hydrogen preparation, storage and application. Among which, hydrogen storage has become the bottleneck technology in the wide spread of hydrogen energy (Felderhoff et al., 2007; Yang J. et al., 2010; Pukazhselvan et al., 2012; Kim et al., 2018).

Hydrogen can be stored as cryogenic liquid, high compression gas or solid-state materials (Yu et al., 2017; Abe et al., 2019). Compared with high cost cryogenic liquid storage and dangerous high compression gas tanks, hydrogen stored in solid-state materials shows easy manipulability temperature, low working pressure (Khafidz et al., 2016; Rusman and Dahari, 2016; Razavi et al., 2019). In the past decades, oceans of materials for hydrogen storage have been investigated, including physical adsorbents (carbon and MOF), complex hydrides (LiBH_4_, LiNH_4_, NaAlH_4_), alloys hydrides (Mg_2_NiH_4_, TiFeH_2_, NaMgH_3_), and metal hydrides (MgH_2_) (Shao et al., 2015; Zhai et al., 2016; Xiao et al., 2017; Chen et al., 2019; Goto et al., 2019; He et al., 2019, 2020; Liu H. et al., 2019, 2020; Song et al., 2019; Jansa et al., 2020; Yao et al., 2020).

Among different solid-state hydrogen storage materials, magnesium hydride (MgH_2_) has been much discussed and holds tremendous hope for storing hydrogen (Bogdanović and Spliethoff, 1990; Norberg et al., 2011; Zhang X. L. et al., 2020). As the sixth abundant metal element in the earth's crust, magnesium is widely distributed in nature. More importantly, MgH_2_ has a high gravimetric capacity of 7.6 wt% (volumetric capacity of 110 g/L) and excellent reversibility. However, the practical application of MgH_2_ has been hindered by the high desorption temperature and poor hydrogen absorption/desorption kinetics caused by high thermal stability (ΔH = 76 kJ/mol) and kinetic barrier (Ea =160 kJ/mol) (Webb, 2015; Peng et al., 2017; Zhou et al., 2019a; Jain et al., 2020).

To overcome above challenges, alloying (Bououdina and Guo, 2002; Liao et al., 2004; Kumar et al., 2013; Xu et al., 2018; Ali et al., 2019), nanostructuring (Chen et al., 2012, 2018; Yu et al., 2014; Sterl et al., 2018), nanoconfinement (Nielsen et al., 2009; Gosalawit-Utke et al., 2011; Jeon et al., 2011; Konarova et al., 2013; He et al., 2015), and doping with catalysts (Su et al., 2016; Sun et al., 2016; Zhang et al., 2018; Pluengphon et al., 2019; Wang et al., 2019) have been adopted to enhance the hydrogen storage properties of MgH_2_. According to recent studies, the transition metals (Ti, Fe, Co, Ni, Mn, Nb, V, Zr, etc.) and their alloys (Shang, 2004; Yavari et al., 2005; Xie et al., 2009; Pighin et al., 2012; Zahiri et al., 2012; Wang et al., 2016) doped in MgH_2_ showed superior modification impacts on the hydrogen storage properties while carbon materials (graphene, carbon nanotubes, and other materials) were proved to enhance the cycling property of MgH_2_. In this work, we systematically review transition metals, their alloys and carbon materials as catalysts to improve the hydrogen storage properties of MgH_2_. In addition, the remaining problems and possible solutions are proposed and discussed.

## Transition Metals and Their Alloys

On the whole, doping transition metals and their alloys into magnesium hydride has been considered as one of the most feasible methods to accelerate the sorption kinetics of MgH_2_. During recent years, numerous transition metals and their alloys have been developed and researched. In this paper, these catalysts are reviewed and classified, presented as monometallic catalysts, binary alloys, ternary and multicomponent alloys and the composites of alloys and carbon materials. Their catalytic effects on hydrogen storage properties of MgH_2_ were summarized in Table 1.

**Table 1:** Dehydrogenation properties of MgH_2_ catalyzed with various materials.

**Samples**	**Non-isothermal dehydrogenation**			**Isothermal dehydrogenation**			**Ea (kJ/mol^**−1**^)**	**References**
	**T_**onset**_ (^**°**^C)**	**T_**peak**_ (^**°**^C)**	**Capacity (wt%)**	**T (^**°**^C)**	**t (min)**	**Capacity (wt%)**		
MgH_2_-10wt%Ni		278.7		250	10	6.1	118	Xie et al., 2009
MgH_2_-10wt%Ti	257	372	6.18				103.9	Wang et al., 2015
MgH_2_-5wt%nano-Fe	177.6	222.6	6.6	300	10	5.44	40.7	Montone et al., 2012
MgH_2_-10wt%nano-ZrMn_2_	182	251	6.7	300	5	6.7	82.2	Zhang et al., 2019a
MgH_2_-10wt%TiMn_2_		377		225	6.7	5.1	82.9	El-Eskandarany et al., 2019a
MgH_2_- TiVMn				270	3	5.5	85.2	Zhou et al., 2013
MgH_2_- Ti_0.4_Cr_0.15_Mn_0.15_V_0.3_				290	30	5.7		Yu et al., 2010
MgH_2_-5wt%FeCoNi@GS	255			290	8.5	6.14	85.1	Singh et al., 2017
MgH_2_-10wt%Zr_0.4_Ti_0.6_Co/5wt%CNTs	200			300	10	6.1	70.5	Zhang L. et al., 2020

## Monometallic Catalysts

### Nickel (Ni)

Monometallic catalysts, especially transition metals (Ershova et al., 2008; Gasan et al., 2012; El-Eskandarany et al., 2016; Tanniru et al., 2020), have shown great catalytic impact on improving the hydrogen storage properties of MgH_2_. Among all the transition metals studied in recent years, nickel has been the mostly adopted catalysts for MgH_2_. As early as 2005, Hanada et al. (2005) mixed purchased MgH_2_ powder with metal Ni by ball milling to get the MgH_2_+nano-Ni composite. Through the thermal desorption mass spectra (TDMS), they found that the hydrogen desorption peak of the Ni doped composite decreased to 260°C, which was much lower than that of pure MgH_2_ (370°C). Although the superior catalytic effect of Ni nanoparticles was confirmed, other factors such as particle size and catalyst amount were also widely researched lately. Xie et al. (2009) studied the hydrogen storage kinetics of the MgH_2_ nanoparticles doped with different concentration of Ni nanoparticles. The DSC curves depicted that the MgH_2_+10 wt% nano-Ni composite could desorb 6.1 wt% hydrogen within 10 min at 250°C. The desorption rate of MgH_2_+nano-Ni composite increased obviously with the increasing amount of catalyst. However, the activation energy of desorption could not be further lowered when the amount of Ni exceeded a certain value by using Kissinger equation. It was concluded that the catalytic effect of Ni could further be increased by reducing the particle size of catalyst and maintaining the hydrogen storage capacity at the same time. Yang W. N. et al. (2010) investigated the size effect of Ni particles on the hydrogen desorption of MgH_2_. The results showed that the MgH_2_ mixed with only 2 at% of fine Ni particles rapidly desorbed hydrogen from 200°C and almost 6.5 wt% hydrogen could be released when the temperature rose to 340°C. Nevertheless, DSC curves showed that the peak temperature of the MgH_2_ + 2Ni_90_ mixture is around 280°C, which was only about10°C lower than those of the MgH_2_ + 2Ni_200_ and the MgH_2_ + 2Ni_100_ composites. They finally concluded that the site density of the catalyst over the MgH_2_ particles but not the particle size was the key factor to improve the hydrogen adsorption kinetics of MgH_2_ after comparing with other references.

### Titanium (Ti)

In comparison with nickel, titanium has also been demonstrated as a good catalyst for MgH_2_. In 1999, Liang et al. (1999a) studied the catalytic mechanism of titanium mainly through XRD results. During the synthesis process of the MgH_2_+5at%Ti composite via mechanical milling, a very stable TiH_2_ phase was formed by reaction of MgH_2_ with Ti. Interestingly, TiH_2_ could be obtained after desorption, which suggests that no decomposition of TiH_2_ phase occurred under the mild desorption condition of 300°C. The desorption curves showed that MgH_2_−5at%Ti composites could desorb hydrogen completely within 1,000 s at 250°C while the ball-milled MgH_2_ released no hydrogen under the same conditions. A lot of work has been done by researchers to further study the de/hydrogenation kinetics and microstructure of MgH_2_-Ti composites. Wang et al. (2015) also prepared the MgH_2_-Ti composite by ball milling, and found that the initial dehydrogenation temperature of the composite to be 257°C, which was 51°C lower than that of pure MgH_2_. The hydrogen capacity could reach 6.18 wt% at the same time. Compared to the sluggish desorption kinetics of pure MgH_2_, the *Ea* for the MgH_2_-Ti sample was 103.9 kJ mol^−1^, about 35.8% lower than that of pure MgH_2_ (161.3 kJ mol^−1^). By analyzing the mechanism it was depicted that elemental Ti reacted with MgH_2_ and formed active TiH_1.971_ during ball-milling, which acted as active species throughout the desorption process. Shao et al. (2011) mainly researched nanostructured Ti-catalyzed MgH_2_ for hydrogen storage. Through EDX measurements, it was discovered that Ti covered the MgH_2_ surface. The DTA curve showed that decomposition of Ti-catalyzed MgH_2_ sample started from below 300°C, which was about 130°C lower than that of the commercial MgH_2_ sample. Hydrogen absorption kinetics of MgH_2_-Ti sample were also investigated and the dehydrogenated sample could absorb 6 mass% hydrogen in <1 h at 257°C, while the commercial MgH_2_ needed an absorption time of about 3 h to reach a capacity of 6 mass% even at 350°C.

### Iron (Fe)

**Figure 1:**
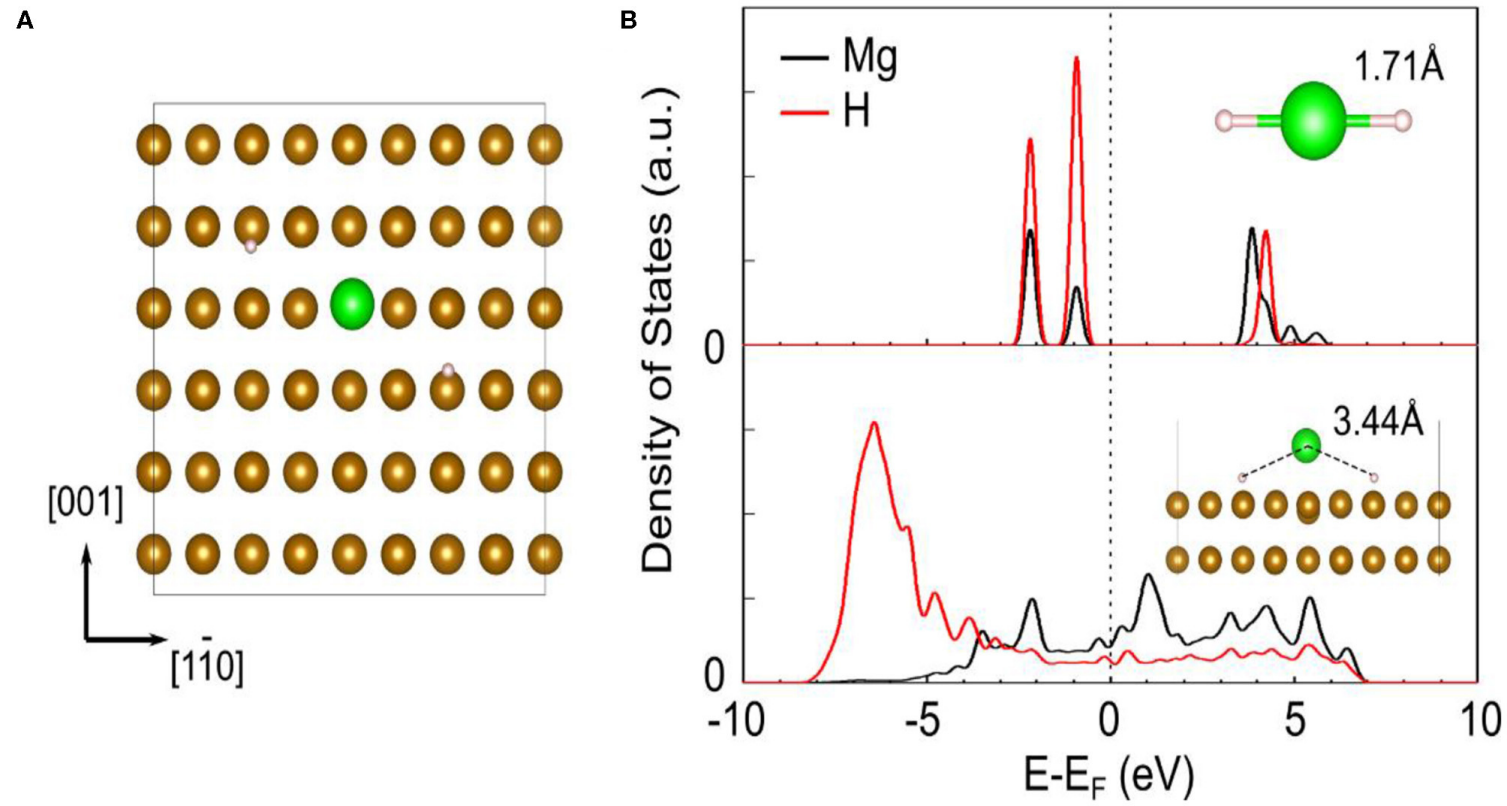
The adsorption geometry of MgH_2_ on Fe (110) surface in top view **(A)**. The brown, green, and white ball represent Fe, Mg, and H, respectively. The projected density of states (PDOS) of Mg and H of MgH_2_ before (upper) and after (lower) adsorption on Fe (110) **(B)**. The Mg-H distances are shown as inset. The vertical dash line represents the Fermi energy. Reproduced from Zhang et al. (2019b) with permission from Elsevier.

As the most common metal element in life, Fe has been widely concerned and studied in recent years. Bassetti et al. (2005) mixed different concentration values of Fe with MgH_2_ by ball milling to explore its catalytic effect. The Mg_2_FeH_6_ phase could be detected when the ball to powder ratio rose to 20:1. They also concluded that the optimum catalyst concentration was around 10 wt% and lower values seemed to be insufficient to avoid the presence of poorly catalyzed regions. DSC curves revealed that about 5 wt% of hydrogen could be released in 600 s at 300°C. Besides the desorption property, the cycling performance and the nano-sized Fe were further studied. Montone et al. (2012) explored the cycling properties of MgH_2_-Fe nanocomposite in 47 cycles at 300°C. Cycling results demonstrated that the maximum storage capacity and the rate of sorption remained stable after the first 10 cycles. They also discovered that the major effect of cycling on particle morphology was the progressive extraction of Mg from the MgO shell surrounding the powder particles. In our previous study (Zhang et al., 2019b), Fe nanosheets obtained by wet-chemical ball milling were introduced in MgH_2_ for the first time. The MgH_2_+5 wt% nano-Fe composite began to release and absorb hydrogen at 182.1 and 75°C, respectively. Moreover, the dehydrogenated composite could absorb 6 wt% H_2_ within just 10 min at 200°C. Cycling experiment depicted that the hydrogen capacity of MgH_2_+5 wt% nano-Fe composite could still maintain at about 5 wt% after 50 cycles. During the first cycle, it could been seen from microstructure pictures that the Fe nanosheets on the surface of MgH_2_ turned to be numerous ultrafine nanoparticles, which could provide more active sites in the following cycling. Further calculation results revealed that the addition of Fe could greatly weaken the Mg-H interaction strength, facilitating the dehydrogenation of MgH_2_ (Figure 1).

### Other Monometallic Catalysts

**Figure 2:**
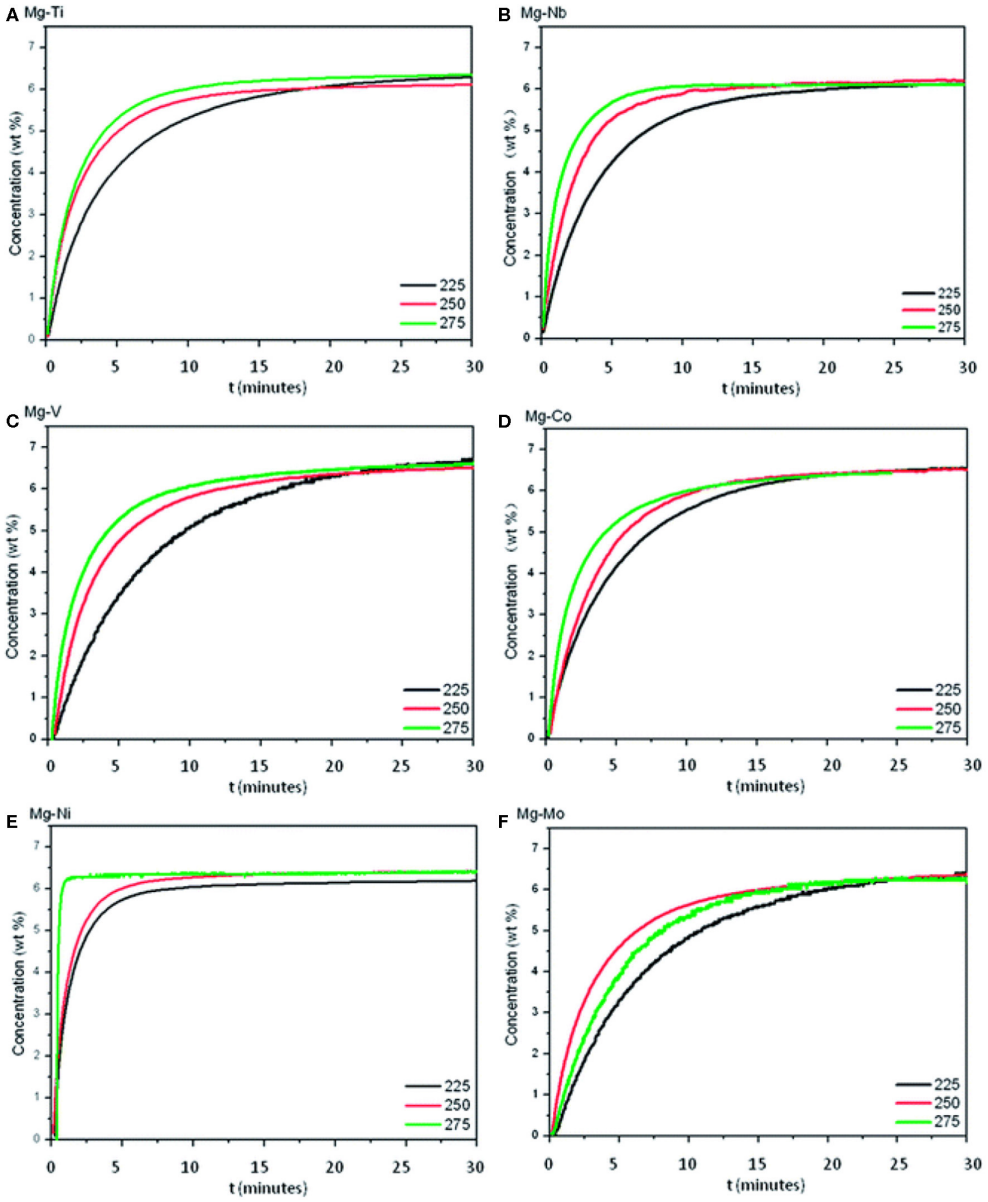
Isothermal dehydrogenation curves of Mg–TM samples at 225, 250, and 275°C. **(A)** Mg-Ti; **(B)** Mg-Nb; **(C)** Mg-V; **(D)** Mg-Co; **(E)** Mg-Ni; **(F)** Mg-Mo. Reproduced from Cui et al. (2014) with permission from Royal Society of Chemistry.

Besides Ni, Ti, and Fe, other monometallic catalysts have also been developed to improve the hydrogen storage properties of MgH_2_. Cui et al. (2014) researched a series of core-shell structured Mg-TM (TM: Ti, Nb, V, Co, Mo, or Ni) nanocomposites by a wet-chemical method. The dehydrogenation performance was ranked as Mg-Ti > Mg-Nb > Mg-Ni > Mg-V > Mg-Co > Mg-Mo (Figure 2). All these composites could release hydrogen at a low temperature of 225°C, which was much lower than that of prepared MgH_2_. Gasan et al. (2012) studied the impacts of 5 wt% of additives (V and Nb) on the hydrogen desorption temperature of MgH_2_. XRD results demonstrated that the addition of V powders had a significant impact on the transformation of Mg into the MgO for the amount of MgO in MgH_2_-V system was higher than other systems, relevant samples were studied by others and this phenomenon needs to be further researched. Also, SEM images verified that the mean particle size of composites was decreased by mechanical milling to micro scale. DSC tests showed that the addition of 5 wt% additives reduced hydrogen desorption temperatures of MgH_2_ by about 40–50°C. Liang et al. (1999b) presented the hydrogen storage properties of MgH_2_+V composite prepared by ball milling. The MgH_2_+5 at% V composite could desorb hydrogen at 200°C and reabsorb hydrogen rapidly even at room temperature, the activation energy of hydrogen desorption was decreased to 62 kJ mol^−1^.

## Binary Alloys

### Zr-Based Binary Alloys

**Figure 3:**
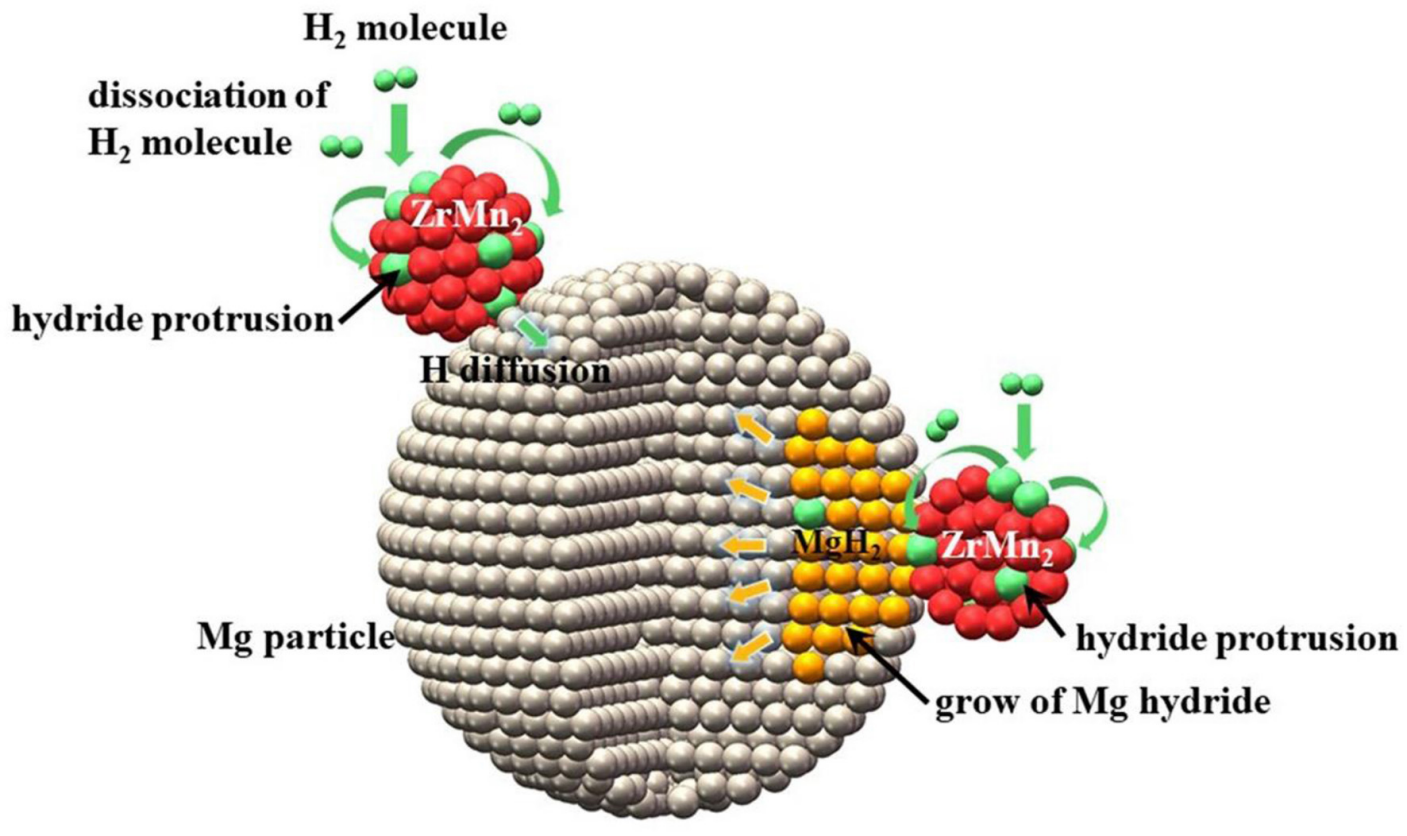
Schematic summary of hydrogenation mechanism of Mg particle catalyzed by ZrMn_2_ nanoportals. Reproduced from Zhang et al. (2019a) with permission from Royal Society of Chemistry.

Recently, many papers reported an interesting strategy for improving hydrogen storage performance of MgH_2_ by using intermetallic compounds of transition metals as catalyst. The corresponding results showed that the absorption/desorption properties of modified MgH_2_ systems should be evidently enhanced by the synergetic effects of both phases. Following above idea, we successfully synthesized ZrMn_2_ nanoparticles ranging from 100 to 300 nm by a facile wet chemical method to explore their catalytic effect on enhancing the hydrogen storage properties of MgH_2_ (Zhang et al., 2019a). The MgH_2_+10 wt% nano-ZrMn_2_ composite began to release hydrogen from 181.9°C and 6.7 wt% hydrogen could be discharged in 5 min at 300°C. Isothermal absorption measurements represented that the dehydrogenated sample could absorb 5.3 wt% hydrogen within 10 min at 100°C under 3 MPa hydrogen pressure. Based on the XRD, TEM and calculation results, the ZrMn_2_ nanoparticles were distributed well on the surface of MgH_2_ and helped weaken Mg-H bond strength, which resulted in the enhanced hydrogen storage performance of MgH_2_ (Figure 3). With the above research experience, we also studied the catalytic effect of ZrCo nanosheets (Zhang et al., 2019c). The modified MgH_2_ composite could desorb almost 6.3 wt% H_2_ within 5 min at 300°C and took up 4.4 wt% H_2_ under 3 Mpa hydrogen pressure in 10 min even at 120°C for doping 10 wt% ZrCo nanosheets. The homogenously distributed ZrCo served as “hydrogen pump” to promote the de/composition of H_2_ molecules, which was the key to decrease the de/hydrogenation temperature of MgH_2_. Cycling performance (10 cycles) revealed that there was an apparent reduction in hydrogen storage capacity. In comparison to our studies, the MgH_2_-ZrNi_5_ nanocomposite system possessed more excellent hydrogen absorption/desorption performance without serious degradation after 600 complete cycles (El-Eskandarany et al., 2017). The prepared MgH_2_-10 wt% ZrNi_5_ sample required 1 and 10 min to absorb and discharge 5.3 wt% H_2_ at 275°C, respectively. Based on the FE-SEM and XRD results, nano-scaled ZrNi_5_ grains were uniformly distributed into the MgH_2_ matrix and ZrNi_5_ particles could create a network of micro channel that extending into the body of metal hydride to provide hydrogen diffusers during the de/hydrogenation processes.

### Ti-Based Binary Alloys

El-Eskandarany et al. (2019a) employed TiMn_2_ compound for improving the de/hydrogenation kinetics of MgH_2_ powders. The 200 h ball milled MgH_2_-TiMn_2_ nanocomposites had a nearly spherical shape with particle size ranging between 100 and 320 nm. DSC analysis presented that this composite could absorb/desorb 5.1 wt% hydrogen within 100 and 400 s at 225°C, respectively. For cycling performance, no obvious degradation in storage capacity was found during the long cyclic-life-time (600 h). FE-SEM micrographs highlighted that TiMn_2_ particles could prevent a serious growth of Mg/MgH_2_ grains, which led to reduced hydrogen uptake/release kinetics. Neto et al. (2017) doped TiFe compound into MgH_2_ and concluded that a fine dispersion could be achieved by increasing milling time or using higher energy ball mill. To attain the best hydrogen kinetics, the sample prepared in the planetary mill for 36 h was the optimum selection and the MgH_2_+ 40 wt%TiFe sample milled for 36 h could release about 3 wt% hydrogen within the first hour. Other Ti-based binary alloys such as TiAl and TiNb have also shown excellent enhancement for catalyzing MgH_2_ (Zhou et al., 2013). Thermo gravimetric analysis and pressure composition temperature (PCT) isothermal tests showed that the MgH_2_-TiAl and MgH_2_-TiNb samples began to desorb hydrogen below 250°C, and the addition of TiAl or TiNb could make the dehydrogenated sample take up hydrogen even at room temperature. In terms of the dehydrogenation reaction, the Mg-H bond would be destabilized by doping with TM elements, which could be confirmed in the theoretical model. The TiAl catalyst illustrated the most effective impact on reducing the activation energy to 65 kJ/mol^−1^ among the Ti-based catalysts. Nevertheless, the Ti intermetallic catalyst did not change the thermodynamic equilibrium pressure of MgH_2_.

### La-Based Binary Alloys

Rare earth elements, especially lanthanides, are considered as one of the most promising catalysts because of their high activity. Many researchers have used La-based binary alloys as catalyst dopants in MgH_2_ to explore the resulting catalytic effects. In 2000, Liang et al. (2000) studied MgH_2_-LaNi_5_ composite and found that Mg, LaH_3_, Mg_2_NiH_4_ were formed during the milling process. The first desorption of mechanically milled MgH_2_-30 wt% LaNi_5_ could release about 4 wt% hydrogen within 150 s at 300°C and the dehydrogenated sample could absorb 3.7 wt% hydrogen in 2,000 s at room temperature. In order to understand the cycling properties of the composite, SEM images manifested that no apparent change in particle size was observed after 20 absorption and desorption cycles. To systematically study the MgH_2_-LaNi_5_ composite, MgH_2_ with different amount of LaNi_5_ were synthesized by Fu et al. (2008). XRD patterns illustrated that the extended milling time of 40 h caused an additional decrease of peak intensity for the materials containing 5 and 15 wt% LaNi_5_, which could be ascribed to the brittleness of LaNi_5_. Further kinetics results showed that the influence of LaNi_5_ on absorption kinetics was more pronounced at lower temperatures. Additionally, other La-based binary alloys could also improve the hydrogen storage properties of MgH_2_. El-Eskandarany et al. (2019b) researched the ball-milled MgH_2_+ nano-LaNi_3_ composite and found a single amorphous phase after 100 h ball-milling. The milled MgH_2_-7 wt% LaNi_3_ sample could discharge 5.6 wt% H_2_ within 37 min at 225°C. For absorption, the dehydrogenated sample could absorb 3.8 wt% H_2_ within 40 min at 125°C. In addition, the MgH_2_-LaNi_3_ sample possessed an extraordinary long cycle-life-time (2,000 h) at 225°C without obvious degradation on its hydrogen storage capacity.

### Other Binary Alloys

Santos et al. (2014) doped a vacuum grade commercial alloy FeNb into MgH_2_ to study its catalytic effect. The MgH_2_-FeNb nanocomposites depicted broaden XRD peaks, which indicated small crystallite size and presence of micro strain. Compared with Fe and Nb, the FeNb exhibited lower activity due to the diverse chemical interfacial energies associated to the nano-interfaces of Mg (MgH_2_)/Fe (or Nb) and Mg (MgH_2_)/FeNb alloy. Recently, we synthesized FeCo nanosheets and confirmed superior catalytic effect on MgH_2_ (Yang et al., 2019). For hydrogen storage performance, DSC curves indicated that the MgH_2_-FeCo composite started to desorb hydrogen from 200°C, which was 150°C lower than that of pure MgH_2_. The dehydrogenated sample could rapidly uptake H_2_ from room temperature and almost 6.7 wt% H_2_ could be absorbed within 1 min at 300°C. Moreover, the MgH_2_-FeCo composite showed excellent cycling performance over 10 cycles. Further TEM and XRD results demonstrated that the FeCo nanoparticles were evenly distributed on the surface of MgH_2_ and functioned as “hydrogen spillover,” which referred that the hydrogen molecules dissociated on the surfaces of FeCo nanosheets and in turn facilitated easy transfer of hydrogen atoms to the surface of Mg particles to generate MgH_2_ during the hydrogenation process. On the other hand, FeCo also effortlessly took up hydrogen atoms from MgH_2_ to form hydrogen molecule, thus remarkably improved the hydrogen storage properties of MgH_2_.

## Ternary and Multicomponent Alloys

### Ti-Based Ternary Alloys

**Figure 4:**
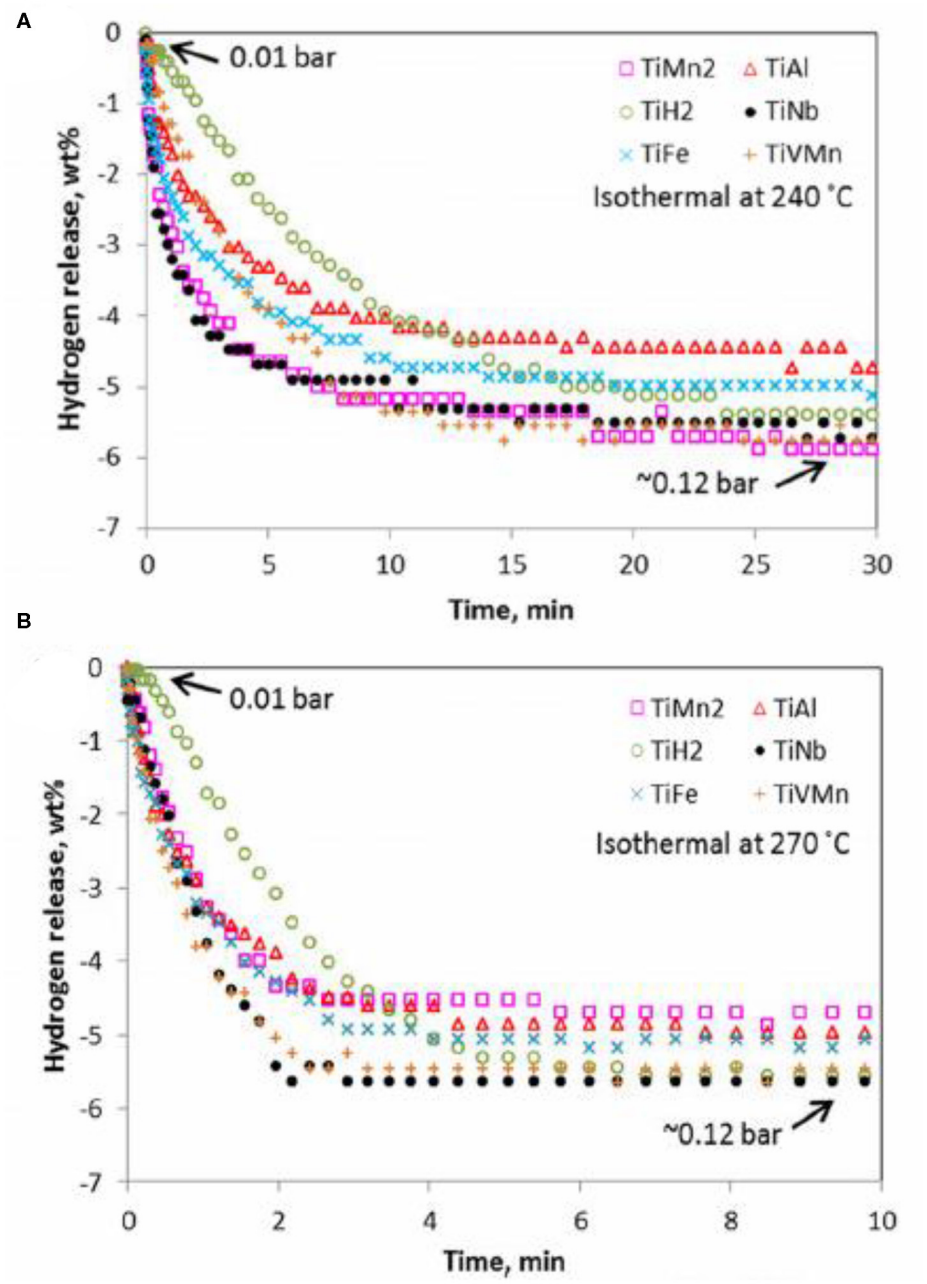
PCT dehydrogenation kinetics for different Ti-based alloy catalyzed magnesium hydrides at 240 °C **(A)** and 270 °C **(B)**. Reproduced from Zhou et al. (2013) with permission from ACS Publications.

Based on the great improvement of the binary alloy, ternary alloys which replace part of the binary alloy with another transition metal have also been concerned in recent years (Hu et al., 2004; Shahi et al., 2013; El-Eskandarany, 2016; Lu et al., 2018). For instance, Zhou et al. (2013) doped TiVMn alloy into MgH_2_ to study its hydrogen storage performance. On the contrast with other Ti-based binary alloys catalysts, the dehydrogenation kinetics of the MgH_2_-TiVMn composite was much better (Figure 4). Moreover, PCT curves also depicted that the addition of TiVMn exhibited the best catalytic effect, which could release more hydrogen under the same condition. The dehydrogenation activation energy was calculated to be 85.2 kJ/mol·H_2_ by OFW model, which was much lower than that of pure MgH_2_. Khodaparast and Rajabi (2015) prepared the MgH_2_+5 at% Ti-Mn-Cr sample by milling the Ti-Mn-Cr alloy produced by melt spinning method with pure MgH_2_. When Ti-Mn-Cr was doped into MgH_2_, the dehydrogenation temperature of the composite reduced from 399 to 345°C, much lower than that of prepared MgH_2_ under the same conditions. Mahmoudi et al. (2011) prepared MgH_2_-5 at% TiCr_1.2_Fe_0.6_ composites at the nanoscale. In comparison to pure MgH_2_, the initial desorption temperature of the MgH_2_-5 at% TiCr_1.2_Fe_0.6_ sample decreased to 241°C and almost 5 wt% hydrogen could be obtained at 300°C. Further, XRD and TEM studies stated that the interface of the TiCr_1.2_Fe_0.6_ alloy with magnesium also acted as active sites for nucleation of the hydride phase, thereby decreasing the nucleation barrier and enhancing the dehydrogenation property.

### Non-Ti-Based Ternary Alloys

Besides Ti-based alloys, other alloys formed by various single transition metals also illustrated their remarkable effect on improving hydrogen storage performance of MgH_2_. Agarwal et al. (2009) studied the catalytic effect of ZrCrNi alloy on hydrogenation properties of MgH_2_. The ZrCrNi alloy was prepared by melting the three pure metals in an arc furnace and then milling with MgH_2_ for 5 h in a SPEX 8,000 mixer-miller to receive the MgH_2_-10 wt% ZrCrNi sample. They also performed 20 cycles of de/hydrogenation to explore the stabilization of kinetics and the achievement of hydrogen capacity. In aspect of de/hydrogenation performance, the composite could quickly desorb and absorb about 90% of its maximum hydrogen capacity within 7 min at 300°C after the 20th cycle. XRD and SEM patterns demonstrated that there was no other phases formed during milling and cycling. Also, the alloy was homogeneously dispersed in the MgH_2_/Mg matrix. To improve hydrogen desorption properties of MgH_2_, Motavalli and Rajabi (2014) prepared the MgH_2_-5 at% Ni_3_FeMn sample by mechanical milling, where the Ni_3_FeMn catalyst was in two states: as-cast and melt-spun ribbon. DTA curves clarified that 30 h mechanically alloyed catalysts in both states could significantly decrease the desorption temperature. MgH_2_-Ni_3_FeMn melt-spun composite could discharge H_2_ in lower temperature due to the ability to improve particle size refinement of MgH_2_ and a more pronounced homogeneous distribution of the alloyed elements. The MgH_2_-5 at% Ni_3_FeMn melt-spun ribbon composite could release 3.39 wt% hydrogen within 1,000 s at 340°C. Zhou et al. (2019b) doped purchased VTiCr into MgH_2_ and demonstrated a reversible capacity of 4 wt% H_2_ between 150 and 350°C for the MgH_2_-VTiCr composite. Besides, the dehydrogenated sample could absorb hydrogen in a low hydrogen pressure of 0.04–0.4 bar. The VTiCr catalyst was uniformly dispersed on the surface of MgH_2_ matrix. VTiCr was deemed as a strong catalyst that provided not only excellent catalytic effect but also offer effective cyclic stability in the sense that the reaction kinetics still remained stable after the 10 cycles.

### Multicomponent Alloys

As mentioned above, single transition metals and their binary and ternary alloys have shown great catalytic effects on MgH_2_-based systems (Haghparast and Rajabi, 2015; El-Eskandarany et al., 2018). Further, studies about multicomponent alloys were also stated, Yu et al. (2010) found that the addition of Ti_0.4_Cr_0.15_Mn_0.15_V_0.3_ alloy could apparently improve the de/absorption properties of MgH_2_. The MgH_2_-Ti_0.4_Cr_0.15_Mn_0.15_V_0.3_ composite began to release hydrogen at 255°C and reached its peak at 294°C, which was much lower than that of unanalyzed MgH_2_. Besides, the dehydrogenated sample could absorb 3.1 wt% H_2_ in 500 min even at 29°C. The cycling results manifested that the dehydrogenation rate increased slowly in the first 20 cycles and then remained stable after 20 cycles. SEM and TEM techniques showed that the Ti_0.4_Cr_0.15_Mn_0.15_V_0.3_ alloy hydride nanoparticles were well-distributed on the surface of MgH_2_. Meena et al. (2018) found that MgH_2_ could desorb H_2_ even at 180°C with the addition of 50 wt% NiMn_9.3_Al_4.0_Co_14.1_Fe_3.6_ alloy. Compared to as-milled MgH_2_ sample, the *Ea* of this composite was lower by about 46.56 kJ/mol. Haghparast and Rajabi (2015) studied the de/hydrogenation kinetics of MgH_2_-TiCrMn_0.4_Fe_0.4_V_0.2_ composite and found that the dehydrogenation temperature of modified MgH_2_ decreased to 378°C, which was lower than that of as-received MgH_2_ (421°C). V_45_Zr_20_Ni_20_Cu_10_Al_3_Pd_2_ powders were doped into MgH_2_ by El-Eskandarany et al. (2018) and found that the desorption temperature of MgH_2_-10 wt% V_45_Zr_20_Ni_20_Cu_10_Al_3_Pd_2_ powders was 308.9°C, which was 116°C lower than that of pure MgH_2_.This prepared nanocomposite possessed superior de/hydrogenation kinetics at relatively low temperature (180°C), absorbing and desorbing 5.5 wt% H_2_ within 200 s.

## Alloys and Carbon Materials

### Alloys and Graphene

**Figure 5:**
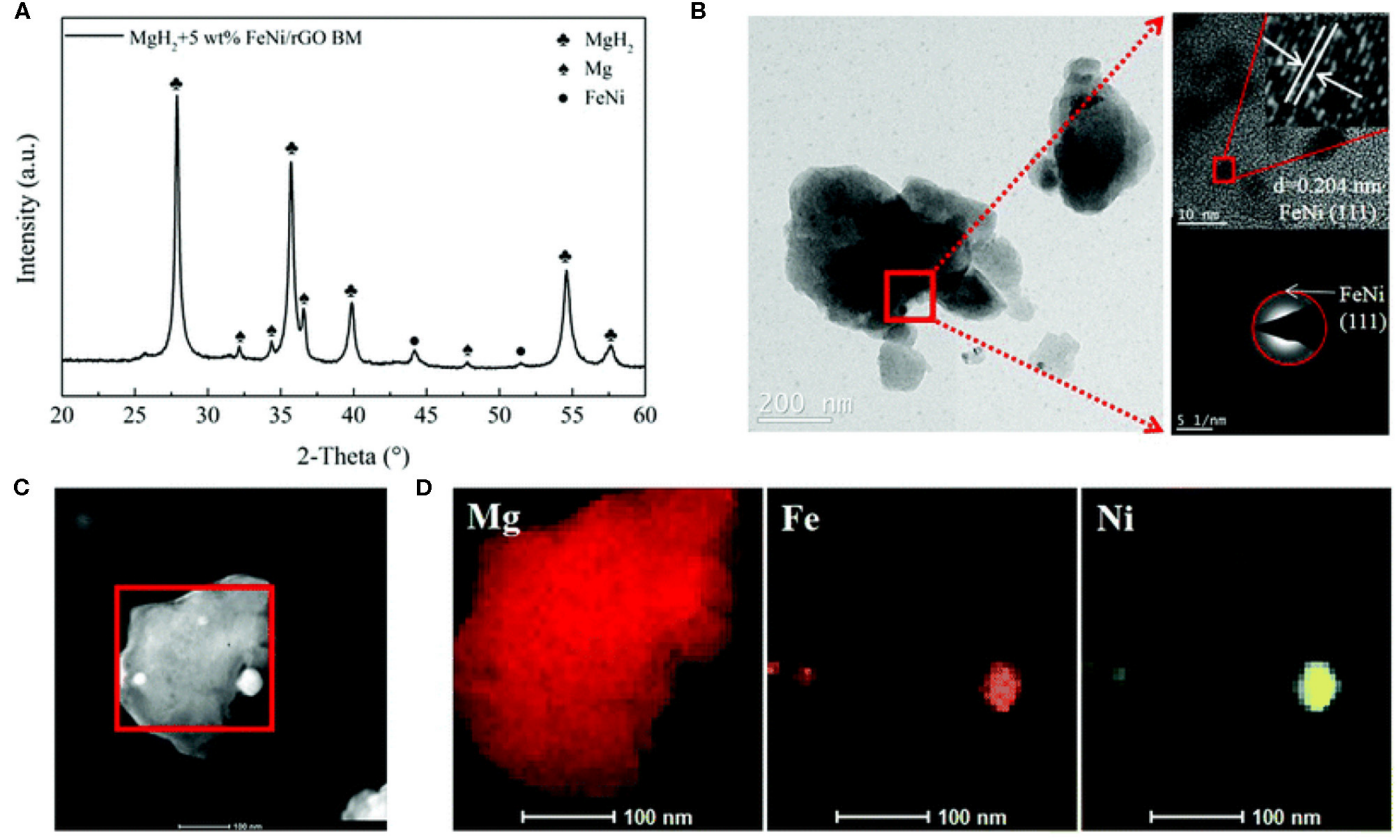
Structural characterization of ball-milled MgH_2_ + 5 wt% FeNi/rGO: **(A)** XRD pattern, **(B)** TEM photograph with the HRTEM image and SAED pattern, and **(C,D)** corresponding EDS spectra with elemental mapping of Mg, Fe, and Ni. Reproduced from Ji et al. (2020) with permission from Royal Society of Chemistry.

All above catalytic materials have shown remarkable improvement on the hydrogen storage performance of MgH_2_, however, stable cycling performance is still the bottleneck for realizing the practical application of MgH_2_. Carbon materials such as graphene and carbon nanotubes, were widely researched and lots of studies have proven that carbon materials are helpful in preserving stable cycling properties (Xia et al., 2015). Hudson et al. (2016) reported that graphene together with Fe nanoclusters could enhance the hydrogen sorption kinetics of MgH_2_. From the TPD and DSC curves, the peak temperature of desorption for MgH_2_+5wt% Fe@G was 281.7°C, lower than that of exhibited peak ball-milled MgH_2_. In addition, the activation energy of MgH_2_+5wt% Fe@G composite was reduced to 119.1 kJ/mol (24% lower than that of ball-milled MgH_2_). Furthermore, TEM confirmed that the grain size of MgH_2_ increased only 15 nm after 6 cycles, displaying a low grain growth rate during cycling due to the addition of graphene. Density functional theory calculations demonstrated that the defect in graphene and the presence of iron clusters at the defect site of graphene played important role in desorbing hydrogen. Ji et al. (2020) prepared FeNi nanoparticles dispersed on reduced graphene oxide nanosheets (FeNi/rGO) and then found that this catalyst played a vital role in improving the hydrogen storage performance of MgH_2_. The MgH_2_-FeNi/rGO sample started to release hydrogen at 230°C and the dehydrogenated sample could absorb 5.4 wt% within 20 min at 125°C. Further investigations proved that FeNi nanoparticles were well distributed on the MgH_2_ surface in the nanoscale range (Figure 5). More importantly, cycling tests exhibited that 6.9 wt% hydrogen capacity was maintained even after 50 cycles. Singh et al. (2017) investigated the catalytic effect of FeCoNi@GS on hydrogen sorption of MgH_2_. The onset desorption temperature of this sample was around 255, 25°C lower than that of FeCoNi catalyzed MgH_2_. The FeCoNi@GS remained stable even after 24 cycles with FeCoNi particles uniformly distributed on the surface of GS.

### Alloys and Carbon Nanotubes

**Figure 6:**
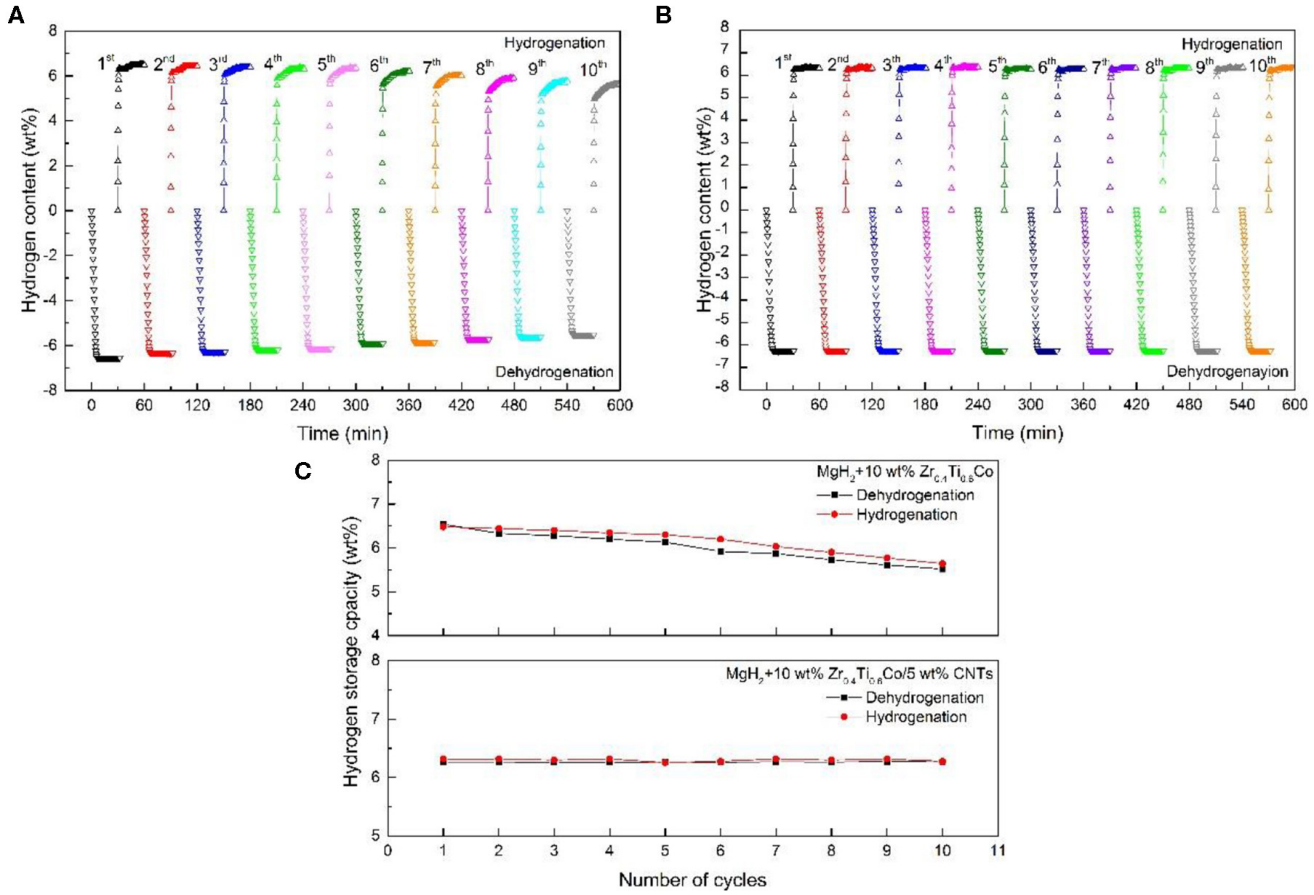
Non-isothermal dehydrogenation/hydrogenation curves of the MgH_2_+10 wt% Zr_0.4_Ti_0.6_Co **(A)**, MgH_2_+10 wt% Zr_0.4_Ti_0.6_Co/5 wt% CNTs **(B)** composites and as a function of cycling and the resulting line chart **(C)**. Reproduced from Zhang L. et al. (2020) with permission from Elsevier.

Carbon nanotubes (CNTs), were widely researched in every field for its small particle size and great microstructure (Luo et al., 2007; Gao et al., 2019; Liu M. et al., 2019). Lillo-Ródenas et al. (2008) demonstrated that the de/hydrogenation performance of MgH_2_ could be strengthened by the addition of different carbon materials. Comparing with other materials, the mixtures involving CNTs and MWCNTs showed the best results to achieve low temperature operation and high hydrogen storage capacity. Ismail et al. (2014) evidenced an apparent catalytic effect of co-doping MgH_2_ with FeCl_3_ and carbon nanotubes on hydrogen storage performance. The CNT-added MgH_2_-FeCl_3_ composite started to release hydrogen at 230, 45°C lower than that of MgH_2_-FeCl_3_. Moreover, the MgH_2_-FeCl_3_/CNT sample could desorb more hydrogen than that of MgH_2_-FeCl_3_ under the same isothermal condition. SEM images confirmed that the CNT was not destroyed after the short milling process and indicated that the sample with CNT appeared to have less agglomeration. It was believed that the presence of the unique structure of the CNTs played a critical role in the improvement of hydrogen storage properties in the MgH_2_-FeCl_3_/CNTs composite. In our recent investigation (Zhang L. et al., 2020), CNTs combined with Zr_0.4_Ti_0.6_Co nanosheets was adopt to strengthen the hydrogen storage properties of MgH_2_. With the addition of Zr_0.4_Ti_0.6_Co sheets, the sorption kinetics were evidently improved while hydrogen capacity was slowly decreasing. Meanwhile, the MgH_2_-Zr_0.4_Ti_0.6_Co/CNTs exhibited no reduction in cycling performance even after 10 cycles after doping CNTs (Figure 6). Deeper structure investigation revealed that particle size of MgH_2_-Zr_0.4_Ti_0.6_Co/CNTs was almost unchanged, contributing to the stable cycling performance.

### Alloys and Other Carbon Materials

Apart from carbon materials mentioned above, other carbon materials also have distinguished effect on ameliorating the de/hydrogenation kinetics of MgH_2_ (Xia et al., 2018). An et al. (2014) reported that the one-dimensional porous Ni@C nanorods modified MgH_2_ performed an excellent hydrogen storage properties. The addition of Ni@C decreased the onset temperature of MgH_2_ to 175°C. Cycling results illustrated no significant loss of hydrogen storage capacity and the MgH_2_-5 wt% Ni@C composite had favorable cycle stability. Chen et al. (2018) reported the mesoporous carbon CMK-3 performed well in enhancing the hydrogen storage properties of MgH_2_. The onset desorption temperature of MgH_2_-10 wt% Ni/CMK-3 was 170°C lower than that of pure MgH_2_ (above 350°C) and the sample could discharge 6 wt% H_2_ even at 295°C. The more fascinating fact was that 3.9 wt% hydrogen was absorbed at 55°C for MgH_2_-Ni/CMK-3 composite. The sample maintained nearly 90.8% of the original de/hydrogenation capacity when cycled for 10 times, indicating that MgH_2_-Ni/CMK-3 had a good cycle stability. Wang et al. (2018) combined graphene oxide-based porous carbon (GC) and TiCl_3_ to improve the reversible kinetics of MgH_2_. The MgH_2_/GC-TiCl_3_ composite could reversibly deliver about 7.6 wt% hydrogen at 300°C within 9 min and the average dehydrogenation rate was several times faster than that of the single catalytic MgH_2_ system. Concerning cycling property, the capacity of the MgH_2_/GC-TiCl_3_ sample was also stable with slower kinetics, owing to the nanoconfinement effect of the ball-milled GC. In a word, graphite and carbon with their derivatives could mainly improve the cycling performance, which results in remarkably enhanced the hydrogen storage properties of MgH_2_.

## Conclusions and Perspectives

To realize the practical application of hydrogen energy, numerous effects still need to be carried out in the coming future. For hydrogen storage materials, magnesium hydride is generally believed as a promising material due to its natural abundance, excellent reversibility, light weight and efficient cost. Among the methods investigated, the transition metals have demonstrated excellent catalytic effect on improving the hydrogen storage properties of MgH_2_. Further studies about alloys based on transition metals are demonstrated to be more effective than the single metal counterparts. In our recent studies, Zr-based alloys and Fe-based alloys were successfully prepared and confirmed to striking improve the de/hydrogenation performance of MgH_2_. Although the transition metals and their alloys have shown superior enhancement on the de/absorption performance of MgH_2_, maintaining good cyclic performance is still a challenge for MgH_2_-based systems. A large number of experiments indicated that carbon materials show excellent effect on maintaining good hydrogen absorption and desorption performance. Our group also demonstrated that carbon nanotubes and reduced graphene oxide together with transition metal alloys can improve the de/hydrogenation kinetics of MgH_2_ while maintain stable cycling properties at the same time. From above review on literature and our own work, we propose the following strategy to further enhance the hydrogen storage properties of MgH_2_: (1) regulate the components of transition metal alloys to its best catalytic effect, (2) make the particle size of the alloys as small as possible, (3) combine alloys and carbon materials to synthetically improve the hydrogen storage properties of MgH_2_. In summary, nanoscale transition metal alloys together with carbon materials would be a promising catalyst for realizing the practical application of MgH_2_.

## Author Contributions

LZ, SS, and JX contributed conception and design of the study. ZS wrote the first draft of the manuscript. XL, FN, and NY wrote sections of the manuscript. All authors contributed to manuscript revision, read and approved the submitted version.

## Conflict of Interest

The authors declare that the research was conducted in the absence of any commercial or financial relationships that could be construed as a potential conflict of interest.
